# Geographical and socioeconomic inequalities in female breast cancer incidence and mortality in Iran: A Bayesian spatial analysis of registry data

**DOI:** 10.1371/journal.pone.0248723

**Published:** 2021-03-17

**Authors:** Shadi Rahimzadeh, Beata Burczynska, Alireza Ahmadvand, Ali Sheidaei, Sara Khademioureh, Forough Pazhuheian, Sahar Saeedi Moghaddam, James Bentham, Farshad Farzadfar, Mariachiara Di Cesare

**Affiliations:** 1 Department of Natural Science, School of Science and Technology, Middlesex University, London, United Kingdom; 2 School of Medicine, Griffith University, Gold Coast, Queensland, Australia; 3 Department of Epidemiology and Biostatistics, School of Public Health, Tehran University of Medical Sciences, Tehran, Iran; 4 ECO College of Insurance, Allameh Tabataba’i University, Tehran, Iran; 5 Non-Communicable Diseases Research Center, Endocrinology and Metabolism Population Sciences Institute, Tehran University of Medical Sciences, Tehran, Iran; 6 School of Mathematics, Statistics and Actuarial Science, University of Kent, Canterbury, United Kingdom; 7 Endocrinology and Metabolism Research Center, Endocrinology and Metabolism Clinical Sciences Institute, Tehran University of Medical Sciences, Tehran, Iran; University of Central Florida, UNITED STATES

## Abstract

**Background:**

In Iran, trends in breast cancer incidence and mortality have generally been monitored at national level. The purpose of this study is to examine province-level disparities in age-standardised breast cancer incidence versus mortality from 2000 to 2010 and their association with socioeconomic status.

**Methods:**

In this study, data from Iran’s national cancer and death registry systems, and covariates from census and household expenditure surveys were used. We estimated the age-standardised incidence and mortality rates in women aged more than 30 years for all 31 provinces in the consecutive time intervals 2000–2003, 2004–2007 and 2008–2010 using a Bayesian spatial model.

**Results:**

Mean age-standardised breast cancer incidence across provinces increased over time from 15.0 per 100,000 people (95% credible interval 12.0,18.3) in 2000–2003 to 39.6 (34.5,45.1) in 2008–2010. The mean breast cancer mortality rate declined from 10.9 (8.3,13.8) to 9.9 (7.5,12.5) deaths per 100,000 people in the same period. When grouped by wealth index quintiles, provinces in the highest quintile had higher levels of incidence and mortality. In the wealthiest quintile, reductions in mortality over time were larger than those observed among provinces in the poorest quintile. Relative breast cancer mortality decreased by 16.7% in the highest quintile compared to 10.8% in the lowest quintile.

**Conclusions:**

Breast cancer incidence has increased over time, with lower incidence in the poorest provinces likely driven by underdiagnoses or late-stage diagnosis. Although the reported mortality rate is still higher in wealthier provinces, the larger decline over time in these provinces indicates a possible future reversal, with the most deprived provinces having higher mortality rates. Ongoing analysis of incidence and mortality at sub-national level is crucial in addressing inequalities in healthcare systems and public health both in Iran and elsewhere.

## Introduction

The second main cause of death globally is cancer which accounts for 25 million new cases and almost 9.6 million deaths (17.1% of total deaths) in 2017 [1]. Estimates suggest that the number of new cancer cases is expected to increase by 20 million annually by 2025 [2], and to double by 2035 [3]. Breast cancer represents approximately 25% of all cancer incidence and about 15% of all cancer deaths among women [4], and it is the most diagnosed cause of cancer death in women worldwide [5, 6]. Globally, breast cancer resulted in almost two million new cases and over 600,000 deaths (2.4% of total female deaths) in 2017 [5].

Several studies have considered the geographical distribution of breast cancer incidence and mortality [7–11]. A large proportion of new female breast cancer cases are now taking place in low-and-middle income countries, with 60% of incidence and 75% of deaths occurring in deprived societies [12]. Moreover, while the age-standardised female breast cancer mortality rate has declined in many high-income countries [4, 13] it is increasing in low- and low-middle income regions [1].

In spite of advances in early detection and treatment for numerous cancers, socioeconomic inequalities persist in cancer incidence and mortality [14]. Although many developed populations have a higher incidence rate of breast cancer [12, 15, 16], this is likely due to better detection [17], with women in poor countries having a higher burden of breast cancer mortality as they are less likely to be screened [18, 19]. This suggests that the high levels of geographical heterogeneity in breast cancer incidence and mortality [20, 21] are partly explained by inequalities in implementation and access to screening or treatment [21].

In Iran, cancer is the third most common cause of death after cardiovascular diseases and motor vehicle accidents [9, 22]. Breast cancer is the most common cancer in women [23], with median age at diagnosis a decade earlier (40 to 50 years old) than in high income countries (over 50 years) [24, 25]. In 2017, the age-standardised incidence rate from breast cancer was 39.8 per 100,000 females (95% UI 31.0,43.4) while the age-standardised death rate was 11.3 deaths per 100,000 females (8.9,11.9), with a percentage change of 128.3 (61.3,189.5) and 38.2 (-0.5,67.8) respectively between 1990 and 2017 [1].

Despite earlier research [9, 26–28], there are no comprehensive studies with reliable data on breast cancer incidence and mortality at subnational level in Iran. Although geographical and subnational disparities are typically ignored in national investigations, they are essential for analysing inequalities and imbalance interventions in healthcare systems [29]. As part of the NASBOD (National and Subnational Burden of Disease, Injuries, and Risk Factors) project in Iran [30], here, we used national cancer registry and death registry data to assess levels of breast cancer incidence and mortality and their association with socioeconomic status (SES) across the 31 Iranian provinces for the period from 2000 to 2010.

## Methods

### Cancer incidence data

Cancer incidence data were collected between 2000 and 2010 by the Iranian Ministry of Health through the National Cancer Registry of Iran, which monitors cancer incidence and includes information on sex, age at diagnosis, province, and district of residence at diagnosis, in addition to the cancer code from the International Classification of Diseases for Oncology [31], as described previously [32, 33]. The first report on all-cancer data, which referred to the various pathology departments in Iran since 1930, dates back to 1960 [34, 35]. Even though this information has been valued among epidemiologists in Iran and in the region [32], it was not designed following cancer register gold standards, hence, its activities were stopped in 1980 and were then resumed in early 2000 using more advanced technology and logistics [33].

The coverage rate for cancer registry was 18% in 2000 (only based on pathology data) [33] but increased to 86% in 2009 (based on pathology and population data) [36]. In this study, we have used data on 48,108 new cases of breast cancer in women aged 30 years old and above, registered in the country between 2000 and 2010 (although data were missing in 2006).

### Mortality data

Mortality data by cause of death at province level were available from the Death Registry System (DRS). Detailed descriptions of the DRS and cleaning methods can be found elsewhere [37]. In addition, all mortality rates have been adjusted by applying the previously calculated completeness rate of registration [38]. The national DRS consists of five sub-datasets, including: DRS data from 1995–2001 and 2001–2004, collected by the Deputy for Research and Technology at provincial level and the Deputy for Public Health at provincial level, respectively; DRS data from 2006–2010, collected by the Deputy for Public Health at provincial and district levels; Behesht-e-Zahra cemetery data from 1995–2010 (Tehran data) and Bagh-e-Rezvan cemetery data from 2007–2010 (Isfahan data) [37]. In this study, we have used data on 17,441 breast cancer deaths in women aged 30 years old and above, registered in the DRS between 2000 and 2010.

### Covariates and populations

Data for incidence and mortality were summarised by age-sex-province-year units. Population data were extracted from the 1996, 2006, and 2011 censuses for each age-sex-province unit [39], with estimates for years between censuses calculated using the population growth formula [40]. In addition, for each year and province the following covariates were included: female urbanisation rate, calculated as the proportion of the female population living in urban areas divided by the total female population; female mean years of schooling (YOS); and wealth index (WI), calculated as the summary measure of 22 household assets extracted from the Household Expenditure and Income Survey (HEIS) (S1 Table and S1 Fig) [39].

The Social Security Insurance (SSI) organisation registry was used to calculate the completeness of the cancer registry as an additional covariate in the model (S1 Table). As treatment for cancer patients is above cost thresholds, insurance organisations have almost 100% coverage for registered cancer patients. Amongst these, SSI with nearly 40% coverage of population in Iran has a comprehensive registry of the financial insurance services for registered cancer patients. Since we assumed that the cancer registry has worked in the same way for other insurance organisations, similar completeness rates have been assumed for all cancer patients, with 22% completeness in 2000 and 75% completeness in 2010, based on the SSI registry [41]. All data were fully anonymized before we accessed them.

### Ethics

The Ethics Committee of the National Institute for Medical Research Development in Iran (IR.NIMAD.REC.1396.192) and the Ethics Committee of the Middlesex University in UK (14142.2020) approved the study protocol.

### Statistical analysis

**Fig 1:**
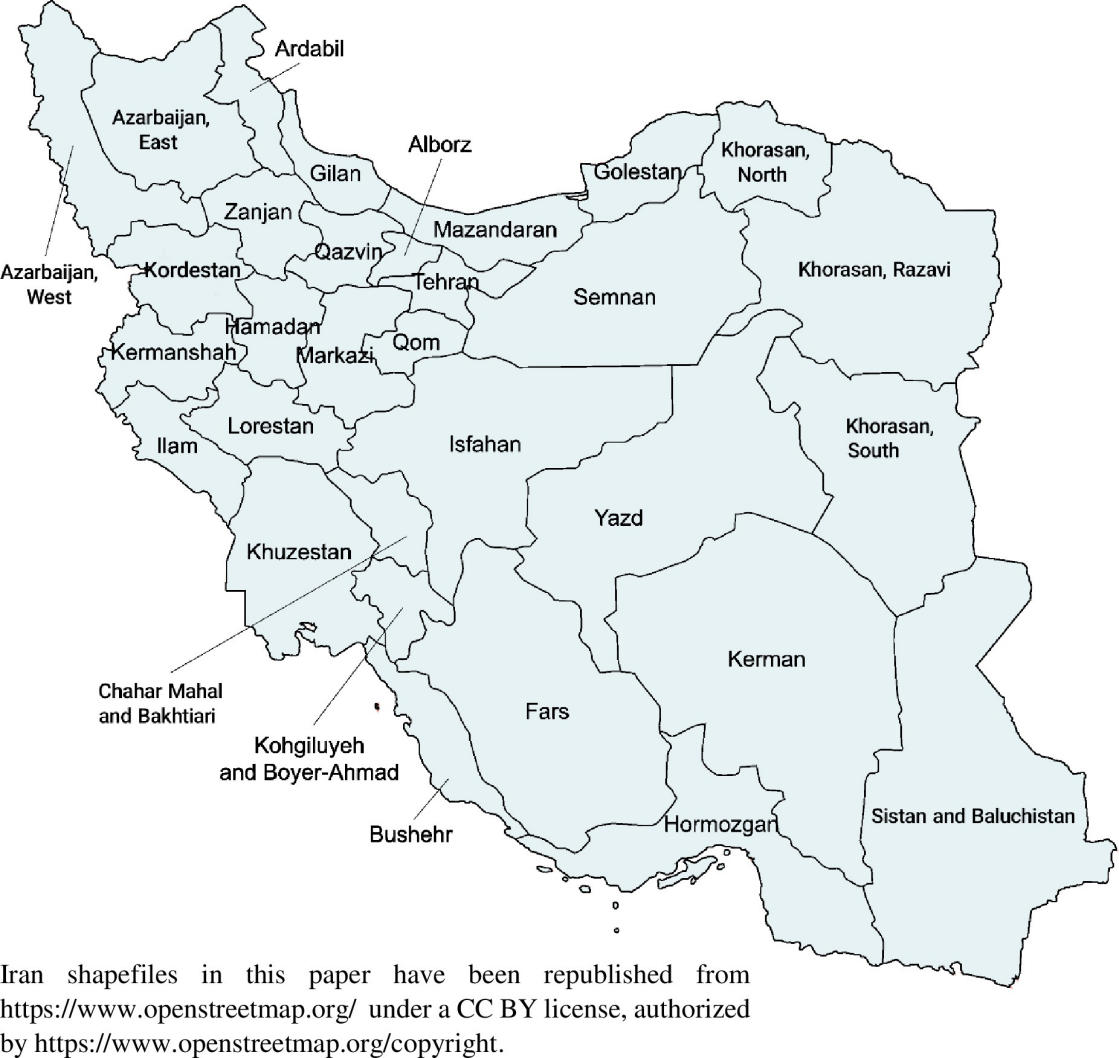
Map of Iran by province.

To analyse geographical inequalities, we estimated age-standardised breast cancer incidence and mortality for the 31 provinces (Fig 1) and three time intervals: 2000–2003, 2004–2007 and 2008–2010. We used the mean national rate for Iran in 2010 in each age group and then multiplied by the population in each province-age group to estimate the expected incidence and deaths. In this study, we applied a Bayesian Poisson spatial model using covariates, which prevented unbalanced estimates and gave proper results in each province. Spatial modelling allows borrowing of information from neighbouring areas, which allows estimation for areas with little or no data [29, 42] (S1 Appendix). The dependence of estimates on data for neighbouring provinces is determined by how stable or unreliable the estimated effects are in each province, and on observed similarities between neighbouring provinces. Applying the Besag, York, and Mollie model, cross-province variance is empirically divided into a spatial component, fitted using a conditional autoregressive prior, and a non-spatial component fitted using a prior with a Gaussian distribution [43, 44]. The model also borrows strength via covariates, which included the incompleteness of the cancer registry, proportion of the province’s population living in urban areas, female mean years of schooling. Household wealth index is used as proxy of the socioeconomic at province level and used as a stratifier for the health outcomes.

In addition, the residuals have been calculated and Moran’s I test [45] has been performed to check that there is no spatial autocorrelation among residuals. The Moran’s I test computes the fitted line slope between the actual residual for each province and the mean residual computed including the neighbouring areas. The obtained Moran’s I coefficient (close to zero) and the associated p-value (p>0.05) indicated that there was no evidence to reject the null hypothesis of no spatial autocorrelation. We therefore conclude that the model allows for spatial patterns appropriately. The inclusion of the three covariates leads to improved model specification according to the Deviance Information Criterion [46].

Our model was fitted in open-source software OpenBUGS version 3.2.3 using the Markov chain Monte Carlo algorithm and R version 3.0.2. This allowed us to make draws from the posterior distribution of the model parameters and to estimate incidence and mortality rates by province and by quintiles of wealth in each province, including the 2.5th and 97.5th percentiles of this distribution as estimates of the lower and upper bounds of credible intervals (CrI), respectively.

## Results

The national age-standardised breast cancer incidence rates (per 100,000 people) in 2000–2003, 2004–2007 and 2008–2010 were respectively 15.0 (95% CrI 12.0,18.3), 22.8 (95% CrI 19.2,26.6) and 39.6 (95% CrI 34.5,45.1), while the mortality rates (per 100,000) were 10.9 (95% CrI 8.3,13.8), 10.3 (95% CrI 8.0,12.9) and 9.9 (95% CrI 7.5,12.5) respectively. The national incidence rate increased by 52% from 2000–2003 to 2004–2007 and by almost 75% between 2004–2007 and 2008–2010. Meanwhile, the percentage reduction in the mean national mortality rate was consistently around 5% between these time periods (S2 and S3 Figs).

**Fig 2:**
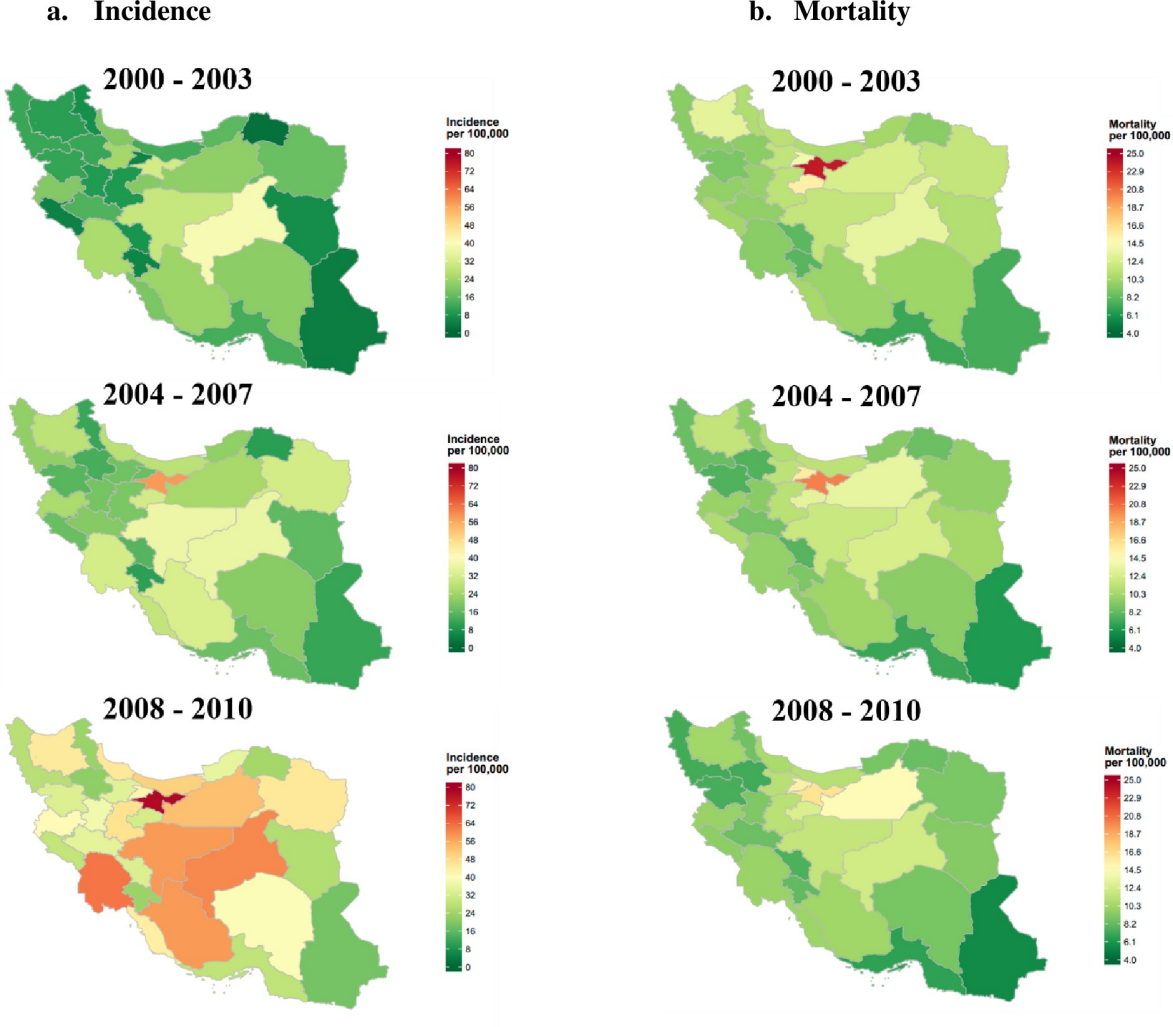
a. Map of posterior age-standardised breast cancer incidence rate by province level for 2000–2003, 2004–2007 and 2008–2010; b. Map of posterior age-standardised breast cancer mortality rate by province level for 2000–2003, 2004–2007 and 2008–2010.

The age-standardised incidence rate for breast cancer was highest in Tehran (78.2 [95% CrI: 75.5,80.9]), Khuzestan (62.8 [95% CrI: 58.4,67.3]), and Yazd (60.5 [95% CrI: 52.2,69.3]) in 2008–2010. In contrast, Sistan and Baluchistan (17.9 [95% CrI: 14.5,21.6]), Zanjan (21.3 [95% CrI: 16.5,26.4]), and Ardabil (22.6 [95% CrI: 18.1,27.5]) were found to have the lowest rates in the same time interval (Fig 2A and Table 1). The breast cancer age-standardised death rate was highest in Tehran (16.2 [95% CrI: 15.0,17.4]), Alborz (15.3 [95% CrI: 12.6,18.2]), and Semnan (14.8 [95% CrI: 10.2,19.9]) in 2008–2010. Meanwhile, Sistan and Baluchistan (5.5 [95% CrI: 3.8,7.4]), Hormozgan (6.7 [95% CrI: 4.4,9.1]), Zanjan (7.2 [95% CrI: 4.8,9.9]) reported the lowest rates (Fig 2B and Table 2).

**Table 1:** Age-standardised breast cancer incidence rates (per 100,000) for females by province and time intervals and percentage change of age-standardised rates (sorted by values in 2008–2010).

	Breast cancer Incidence rate (CrI)			
	2000–2003	2004–2007	2008–2010	Percentage change between 2000–03 and 2008–10 (%)
**Tehran**	31.3 (29.6,33.0)	58.3 (56.1,60.5)	78.2 (75.5,80.9)	149.9 (145.0,155.2)
**Khuzestan**	25.2 (22.4,28.1)	31.5 (28.6,34.5)	62.8 (58.4,67.3)	149.6 (139.7,160.8)
**Yazd**	38.2 (31.6,45.4)	36.2 (30.3,42.6)	60.5 (52.2,69.3)	58.3 (52.4,65.2)
**Isfahan**	29.1 (26.5,31.8)	36.3 (33.6,39.0)	58.4 (54.7,62.3)	101.0 (95.9,106.8)
**Fars**	23.0 (20.5,25.5)	32.3 (29.6,35.1)	58.4 (54.5,62.5)	154.2 (144.8,165.5)
**Semnan**	21.2 (15.5,27.8)	24.6 (18.6,31.1)	53.0 (43.3,63.3)	149.6 (127.7,180.3)
**Mazandaran**	12.4 (10.4,14.6)	27.3 (24.5,30.2)	50.1 (46.0,54.4)	303.2 (272.6,341.8)
**Markazi**	8.4 (6.1,11.2)	18.8 (15.4,22.5)	46.9 (40.9,53.1)	457.1 (375.9,575.7)
**Khorasan, Razavi**	16.7 (14.9,18.7)	31.3 (28.9,33.7)	45.5 (42.4,48.7)	171.8 (160.7,184.4)
**Gilan**	19.9 (17.2,22.7)	27.3 (24.3,30.4)	45.3 (41.1,49.7)	127.8 (118.7,139.1)
**Azarbaijan, East**	9.7 (8.1,11.4)	27.5 (24.9,30.3)	45.2 (41.5,48.9)	365.6 (328.1,414.4)
**Bushehr**	18.8 (13.7,24.5)	28.7 (22.9,35.1)	43.8 (36.1,51.9)	133.2 (112.3,163.4)
**Alborz**	4.8 (3.2,6.6)	17.0 (14.2,19.9)	42.4 (37.9,47.2)	793.5 (620.8,1082.8)
**Kermanshah**	19.9 (16.5,23.5)	25.2 (21.7,28.9)	41.0 (36.1,46.1)	105.9 (96.3,118.3)
**Kerman**	20.7 (17.5,24.1)	20.4 (17.5,23.5)	39.1 (34.8,43.6)	88.6 (81.0,98.6)
**Hamadan**	8.7 (6.5,11.3)	18.3 (15.2,21.6)	37.2 (32.4,42.3)	326.3 (275.6,397.7)
**Golestan**	15.3 (12.0,18.7)	21.5 (18.0,25.3)	35.2 (30.3,40.3)	130.6 (115.7,152.6)
**Lorestan**	13.8 (10.7,17.1)	20.0 (16.6,23.7)	34.7 (29.8,39.8)	151.7 (132.6,177.6)
**Qazvin**	23.8 (18.9,29.1)	17.6 (13.9,21.7)	34.1 (28.5,40.2)	43.5 (38.2,50.7)
**Chahar Mahal and Bakhtiari**	7.6 (4.5,11.3)	14.5 (10.6,18.9)	33.2 (26.6,40.4)	336.1 (258.8,496.0)
**Kordestan**	10.8 (7.9,14.0)	14.6 (11.5,17.9)	33.1 (28.0,38.3)	205.7 (173.6,253.7)
**Qom**	20.2 (15.6,25.4)	31.2 (25.7,37.1)	31.8 (25.9,38.0)	57.0 (49.4,66.5)
**Ilam**	4.6 (1.9,8.4)	16.8 (11.5,22.8)	28.6 (21.1,37.0)	524.0 (342.9,994.8)
**Hormozgan**	12.8 (9.3,16.6)	16.9 (13.3,21.0)	28.1 (23.1,33.5)	120.3 (101.5,148.0)
**Azarbaijan, West**	11.3 (9.2,13.5)	21.7 (18.9,24.5)	27.0 (23.8,30.4)	138.9 (124.7,157.9)
**Khorasan, South**	6.6 (3.4,10.4)	15.3 (10.8,20.5)	26.0 (19.7,33.2)	293.2 (220.1,483.4)
**Khorasan, North**	2.0 (.6,3.9)	9.7 (6.5,13.4)	24.0 (18.4,30.2)	1111.1 (673.1,3013.6)
**Kohgiluyeh and Boyer-Ahmad**	5.2 (2.1,9.0)	10.0 (6.2,14.3)	22.9 (16.4,30.0)	345.0 (234.6,684.2)
**Ardabil**	7.0 (4.7,9.7)	11.4 (8.5,14.6)	22.6 (18.2,27.5)	223.0 (183.1,288.7)
**Zanjan**	11.3 (7.9,15.1)	13.1 (9.7,17.0)	21.3 (16.5,26.4)	88.3 (75.2,110.7)
**Sistan and Baluchistan**	3.8 (2.2,5.6)	11.1 (8.7,13.8)	17.9 (14.5,21.6)	374.3 (286.7,563.9)

**Table 2:** Age-standardised breast cancer mortality rates (per 100,000) for females by province and time intervals and percentage change of age-standardised rates (sorted by values in 2008–2010).

	Breast cancer Mortality rate (CrI)			
	2000–2003	2004–2007	2008–2010	Percentage change between 2000–03 and 2008–10 (%)
**Tehran**	23.8 (22.3,25.3)	20.2 (18.9,21.5)	16.2 (15.0,17.4)	-31.9 (-32.7,-31.2)
**Alborz**	13.8 (11.0,16.7)	15.7 (13.1,18.5)	15.3 (12.6,18.2)	10.9 (9.0,14.5)
**Semnan**	12.4 (8.2,17.2)	13.3 (9.1,17.8)	14.8 (10.2,19.9)	19.4 (15.7,24.4)
**Qom**	15.4 (11.4,19.9)	13.1 (9.8,16.9)	12.5 (9.2,16.3)	-18.8 (-19.3,-18.1)
**Yazd**	13.3 (9.7,17.2)	12.4 (9.2,15.8)	12.1 (8.9,15.6)	-9.0 (-9.3,-8.2)
**Isfahan**	11.5 (9.9,13.2)	11.8 (10.3,13.4)	11.6 (10.0,13.2)	.9 (.0,1.0)
**Markazi**	11.2 (8.6,14.2)	11.1 (8.7,13.8)	11.2 (8.5,14.0)	.0 (-2.1,-1.2)
**Qazvin**	11.4 (8.4,14.7)	11.2 (8.4,14.3)	11.1 (8.2,14.3)	-2.6 (-2.7,-2.4)
**Mazandaran**	10.7 (8.9,12.7)	11.3 (9.6,13.1)	11.0 (9.1,12.9)	2.8 (1.6,2.2)
**Gilan**	11.1 (9.2,13.2)	10.8 (9.0,12.7)	10.6 (8.6,12.6)	-4.5 (-6.5,-4.5)
**Azarbaijan, East**	13.1 (11.2,15.1)	11.5 (9.8,13.2)	10.2 (8.6,12.0)	-22.1 (-23.2,-20.5)
**Fars**	10.0 (8.4,11.6)	10.1 (8.7,11.7)	10.2 (8.6,11.8)	2.0 (1.7,2.4)
**Bushehr**	10.4 (7.1,14.3)	9.5 (6.6,13.0)	10.1 (6.9,13.6)	-2.9 (-4.9,-2.8)
**Khuzestan**	9.4 (7.8,11.2)	9.8 (8.2,11.4)	9.9 (8.3,11.7)	2.1 (1.7,2.7)
**Kermanshah**	9.7 (7.5,12.1)	9.9 (7.8,12.2)	9.9 (7.7,12.3)	5.3 (4.5,6.4)
**Ilam**	10.3 (6.1,15.1)	10.7 (6.7,15.3)	9.6 (5.9,14.0)	-6.8 (-7.3,-3.3)
**Khorasan, South**	10.9 (7.0,15.2)	10.3 (7.0,14.2)	9.3 (5.9,13.3)	-14.7 (-15.7,-12.5)
**Hamadan**	9.8 (7.6,12.3)	9.4 (7.3,11.7)	9.1 (7.0,11.4)	-7.1 (-7.9,-7.3)
**Khorasan, Razavi**	11.6 (10.1,13.2)	9.7 (8.4,11.0)	8.9 (7.6,10.3)	-23.3 (-24.8,-22.0)
**Kerman**	10.1 (8.0,12.4)	9.5 (7.6,11.5)	8.9 (7.0,10.8)	-14.6 (-14.5,-14.1)
**Golestan**	10.3 (7.9,13.1)	9.3 (7.0,11.6)	8.8 (6.7,11.2)	-12.9 (-12.9,-12.5)
**Ardabil**	10.8 (7.9,14.0)	9.5 (7.0,12.3)	8.6 (6.1,11.4)	-20.4 (-22.8,-18.6)
**Lorestan**	9.7 (7.3,12.3)	8.7 (6.6,10.9)	8.4 (6.3,10.8)	-10.6 (-12.9,-11.5)
**Khorasan, North**	9.4 (6.2,13.1)	8.5 (5.8,11.6)	8.4 (5.4,11.6)	-13.4 (-13.7,-12.2)
**Kohgiluyeh and Boyer-Ahmad**	7.9 (4.5,11.9)	9.0 (5.7,12.9)	8.1 (4.8,11.9)	2.5 (.0,6.7)
**Chahar Mahal and Bakhtiari**	8.0 (5.0,11.3)	7.8 (5.1,10.8)	7.6 (4.9,10.7)	-5.0 (-5.3,-4.0)
**Kordestan**	8.9 (6.5,11.6)	7.7 (5.6,9.9)	7.4 (5.2,9.7)	-16.9 (-20.0,-16.4)
**Azarbaijan, West**	9.4 (7.6,11.5)	8.4 (6.7,10.1)	7.3 (5.6,9.0)	-22.3 (-26.3,-21.7)
**Zanjan**	9.5 (6.6,12.7)	7.7 (5.3,10.3)	7.2 (4.8,9.9)	-24.2 (-27.3,-22.0)
**Hormozgan**	7.1 (4.7,9.7)	7.0 (4.8,9.4)	6.7 (4.4,9.1)	-5.6 (-6.4,-6.2)
**Sistan and Baluchistan**	7.3 (5.2,9.7)	6.3 (4.5,8.2)	5.5 (3.8,7.4)	-24.7 (-26.9,-23.7)

Provinces with the highest percentage of age-standardised incidence rates between 2000–2003 and 2008–2010 were Khorasan, North (1111.1% [95% CrI: 673.1,3013.6]), Alborz (793.5% [95% CrI: 620.8,1082.8]), and Ilam (524% [95% CrI: 342.9,994.8]). In contrast, Qazvin (43.5% [95% CrI: 38.2,50.7]), Qom (57.0% [95% CrI: 49.4,66.5]) and Yazd (58.3% [95% CrI: 52.4,65.2]) had the lowest percentage of incident rates. Provinces with the greatest significant increasing trends in age-standardised death rates were Semnan (19.4% [95% CrI: 15.7,24.4]), Alborz (10.9% [95% CrI: 9.0,14.5]), and Khuzestan (5.3% [95% CrI: 4.5,6.4]). Meanwhile, Tehran (-31.9% [95% CrI: -31.2 to -32.7]), Sistan and Baluchistan (-24.7% [95% CrI: -23.7 to -26.9]), and Zanjan (-24.2% [95% CrI: -22.0 to -27.3]) experienced the highest decreasing trend in death rate from 2000–2003 to 2008–2010 (Tables 1 and 2).

**Fig 3:**
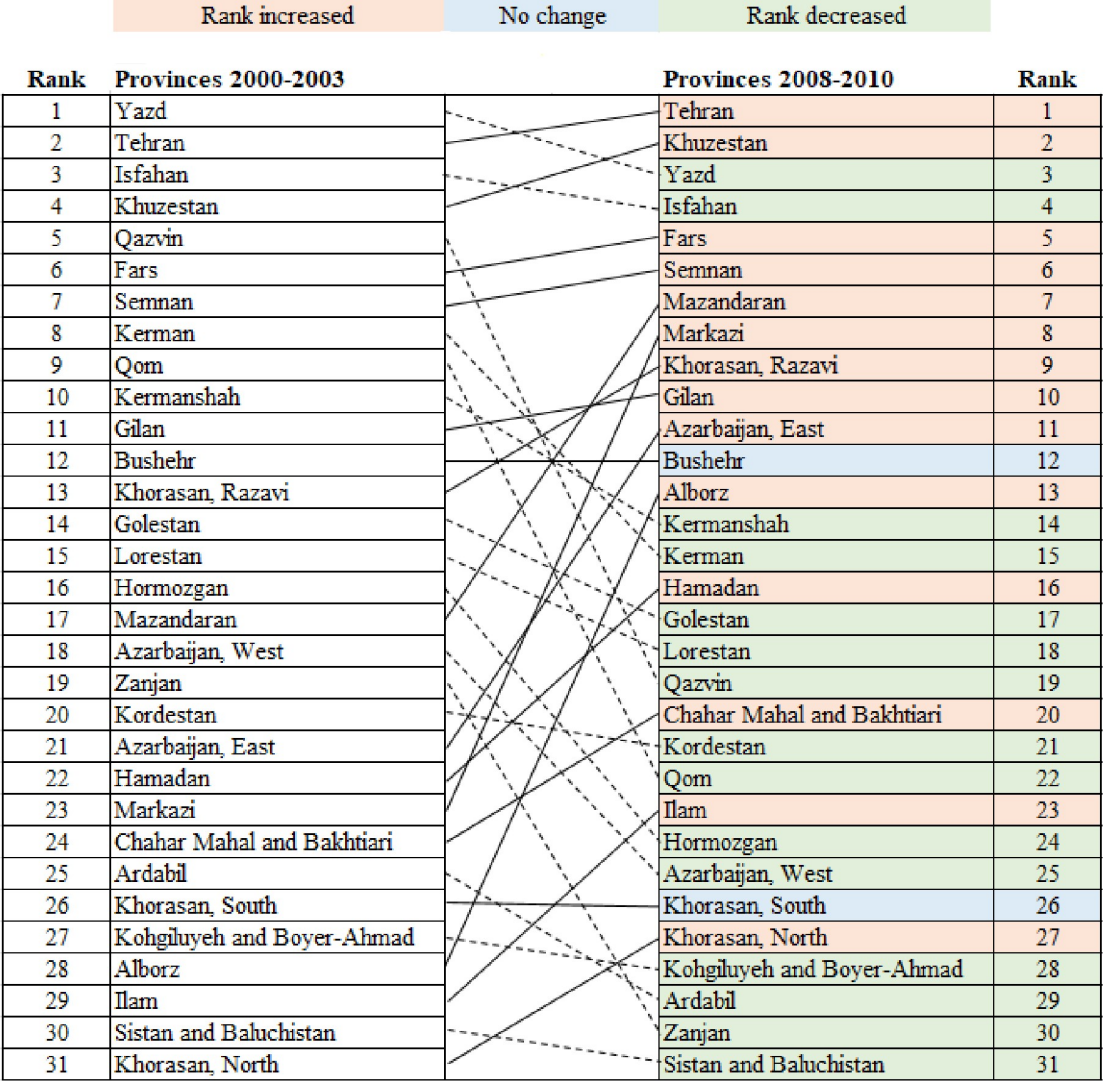
Provinces ranked by age-standardised incidence rate for 2000–2003 and 2008–2010. Dotted and solid lines show decrease and increase of rank, respectively.

**Fig 4:**
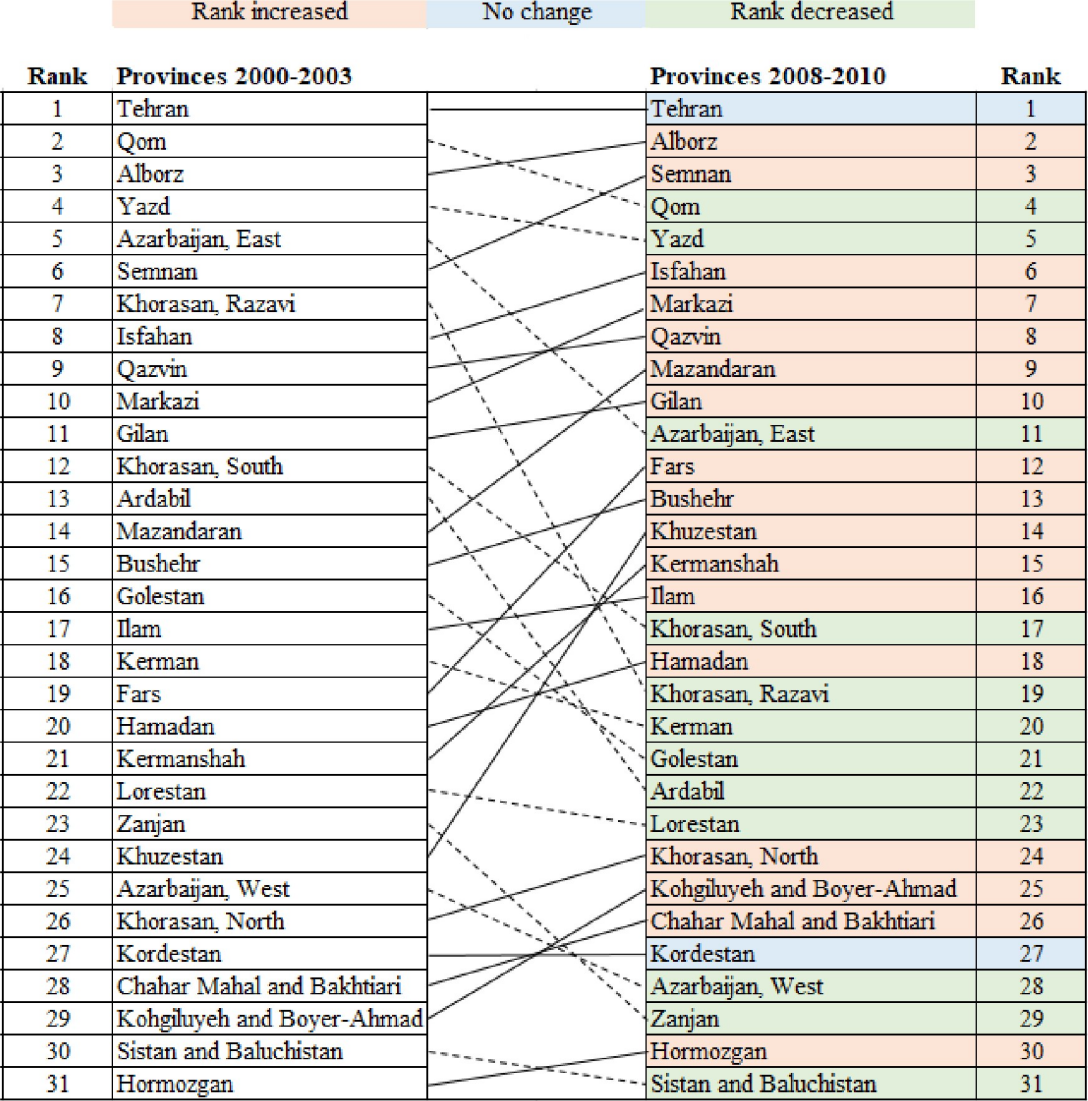
Provinces ranked by age-standardised mortality rate for 2000–2003 and 2008–2010. Dotted and solid lines show decrease and increase of rank, respectively.

Arrow diagrams (Fig 3) indicate that Yazd province had the highest incidence rate in 2000–2003 while in 2008–2010 Tehran was in the upper level. However, Tehran as the most populous city in Iran, had the highest mortality rates in 2000–2003 and 2008–2010 (Fig 4).

**Fig 5:**
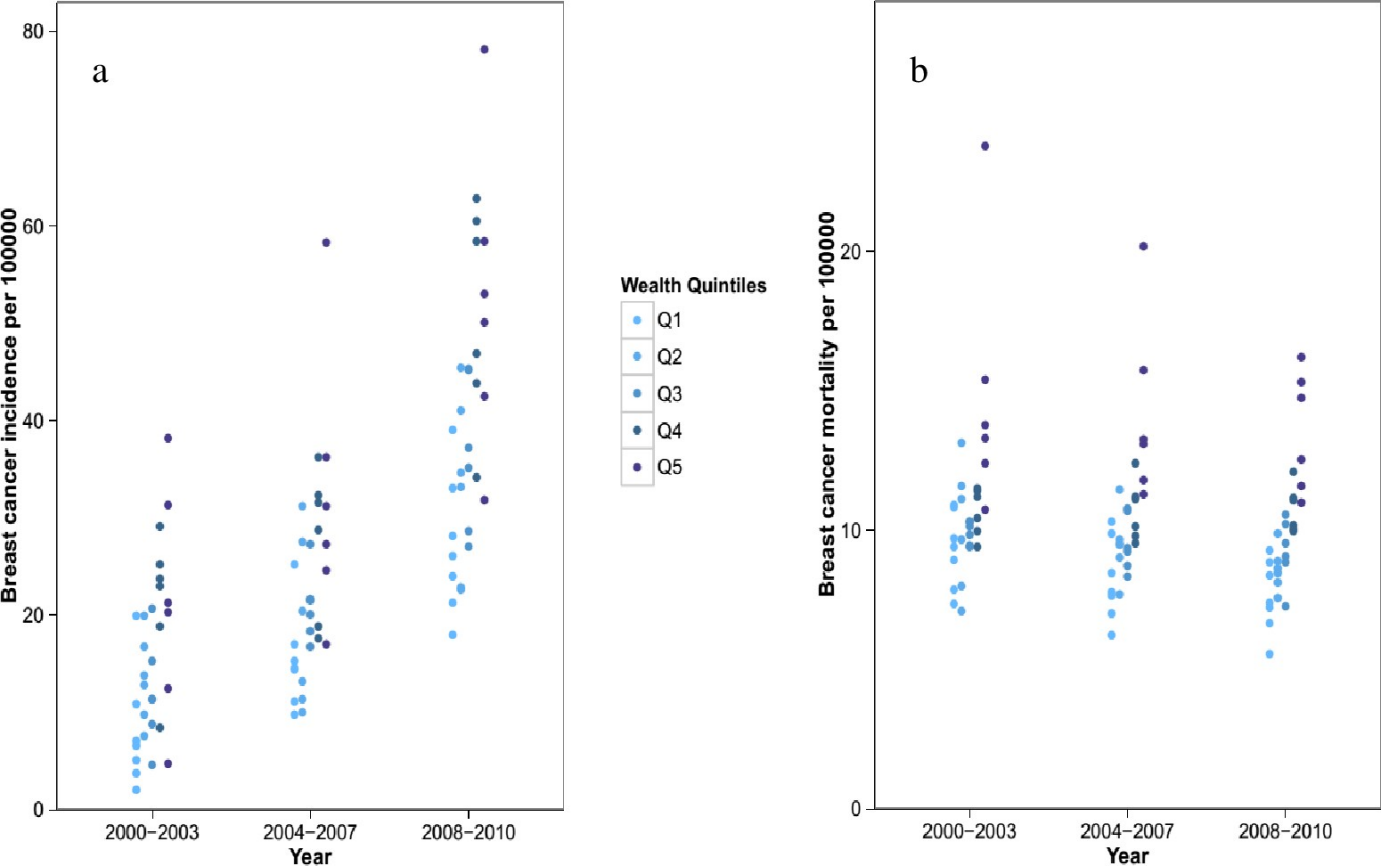
Breast cancer incidence (a) and mortality (b) rate by province arranged by quintiles of province wealth. Each dot represents the posterior mean of incidence and mortality for one province. The darkest colour show the wealthiest quintile and the lightest colour the most-deprived quintile.

When grouped by wealth index quintiles (Fig 5 and Table 3), provinces in the highest quintile had higher levels of breast cancer incidence in 2000–2003 (average from 7.0 per 100,000 people in the lowest quintile to 24.1 per 100,000 people in the highest quintile), 2004–2007 (15.7,32.0) and 2008–2010 (30.7,48.8). Similarly, provinces in the highest quintile had greater levels of breast cancer mortality in 2000–2003 (mean from 9.3 per 100,000 people in the lowest quintile to 15.0 per 100,000 people in the highest quintile), 2004–2007 (8.6,13.5) and 2008–2010 (8.3,12.5). While the national mortality rate is decreasing, the reduction in wealthier provinces is much greater than in less wealthy provinces.

**Table 3:** Age-standardised breast cancer incidence and mortality rates (per 100,000) by province wealth index quintile.

		Poorest quintile	Q2	Q3	Q4	Q5
Incidence	2000–2003	7.0 (4.5,9.9)	12.7 (9.8,16.0)	14.1 (10.9,17.5)	18.3 (15.5,21.2)	24.1 (20.3,28.3)
	2004–2007	15.7 (12.1,19.6)	17.7 (13.6,22.2)	22.3 (19.4,25.5)	27.4 (24.0,31.0)	32.0 (28.2,36.0)
	2008–2010	30.7 (25.2,36.7)	31.8 (26.3,37.8)	41.0 (36.1,46.2)	47.3 (42.6,52.2)	48.8 (43.8,54.0)
Mortality	2000–2003	9.3 (6.4,12.5)	9.3 (6.7,12.1)	10.5 (8.1,13.3)	10.7 (8.7,12.9)	15.0 (12.1,18.2)
	2004–2007	8.6 (6.1,11.4)	9.0 (6.3,12.0)	10.1 (8.2,12.2)	10.9 (8.7,13.2)	13.5 (11.1,16.1)
	2008–2010	8.3 (5.7,11.3)	9.0 (6.3,11.9)	9.5 (7.3,11.9)	10.4 (8.2,12.6)	12.5 (10.1,15.1)

## Discussion

The current study reports the age-standardised breast cancer incidence and death rates across 31 provinces in Iran between 2000 and 2010. Nationally, there was a substantial rise in age-standardised incidence rates, while age-standardised death rates were identified to have a decreasing trend. Several provinces, such as Semnan, Alborz, Khuzestan, Mazandaran, Kohgiluyeh and Boyer-Ahmad, Kermanshah, Fars, and Isfahan showed different patterns with substantial increase in death age-standardised rates.

We tried to compare our results with previous studies; however, no studies were found for a direct comparison at the year-province-specific level of incidence and mortality rates. While our study found that all the aforementioned provinces had increasing trends in age-standardised incidence rate during 2000–2010, a previous study showed estimated overall incidence rate of breast cancer had a smooth decreasing pattern in Iran in 2004–2008 [28]. Although the trend of age-standardised mortality rate of breast cancer increased dramatically during 1995 to 2004 [47] and 2006–2010 [48], our findings show a declining trend in mortality rate between 2000 and 2010.

Based on available studies [26, 49] for 30 provinces from 2004 to 2009, Gilan and Azerbaijan, East had the highest risk and Kohgiluyeh and Boyer-Ahmad had the lowest risk of breast cancer incidence. Likewise, another study [50] reported that the age-standardised rate of breast cancer in Azerbaijan, East was higher in 2006–2007, compared to that of Ardabil, which had the lowest rate. However, we found Tehran, Yazd, Khuzestan and Isfahan have the highest age-standardised breast cancer incidence rate and Sistan and Baluchistan has the lowest rate. Khorasan, Razavi and Golestan notably experienced the steepest increasing trend in breast cancer incidence from 2004 to 2008 among 30 provinces [28], while our findings show Khorasan, North; Alborz and Ilam have the greatest percentage change of incidence rate from 2000–2010.

Our results suggest high levels of geographical heterogeneity in breast cancer incidence and mortality across Iranian provinces. All provinces in our study have age-standardised incidence and mortality rates well above the cumulative probability of breast cancer incidence and death for individuals aged 15–79 years at the national level in Iran in 2000 (2.4 per 100,000 women and 0.7, respectively) and 2010 (2.8 and 0.7, respectively) estimated by the Global Burden of Disease study [10].

Previous research shows that cancer incidence and mortality in Asian countries have respectively a positive and a negative correlation with the country’s level of development measured by the Human Development Index [7]. In particular, greater financial development and larger and more complex cancer prevention policies are associated with lower mortality within each major income level [18]. Moreover, female age-standardised incidence rate decreased in high socio-demographic index (SDI) countries but increased in the other SDI quintiles from 2007 to 2017 [5]. In Iran, a direct and substantial association was also found between the incidence of breast cancer and the Human Development Index [26]. These findings are similar to our results in which higher levels of breast cancer incidence are observed across provinces with higher level of wealth index. This could be explained by increasing life expectancy, urbanisation, higher exposure to risk factors, delayed childbearing, a higher rate of screening, and better cancer registries [15]. Furthermore, high-income countries are characterised by diets higher in fats and also by higher levels of obesity, with both factors associated with higher risk of postmenopausal breast cancer (12–13% increase in risk per 5 kg/m^2^) [51, 52].

Although we anticipated observing a lower mortality rate in the least deprived provinces in our study, most provinces with higher rates of incidence and mortality were in higher quintiles of the wealth index. However, the slope of mortality reduction over time among provinces in the wealthiest quintile is larger than that observed in the poorest quintile, which suggests a possible reverse association in coming years, agreeing with other existing studies [53, 54].

It appears that the high incidence rates observed in our study are likely owing to higher breast screening in the last time interval of study (2008–2010) compared to the first time interval (2000–2003) especially among those groups who were in wealthier areas [55, 56]. The increasing completeness percentage of cancer registry over time in Iran (S1 Table) may also have played an important role in the increases in reported numbers of new cases. Nevertheless, despite the potential benefits of screening, previous findings demonstrate that breast cancer screening usage rate among Iranian women is low (1.3% to 30.5%) [57]. For instance, screening rate in the North of Iran varied from 21.7% of women in Mazandaran to only 15.7% of women in Gilan [58, 59]. Also, in the South of Iran, only 1.3% of the women had a mammography screening at any point in their lifetime [60]. This suggests that taking full advantage of female screening participation in our community must be considered as a fundamental priority.

These efforts have some limitations. Firstly, the increase in coverage rate of the cancer registry over the period of analysis may impact on the interpretation of the time trends. However, we have addressed this issue by including coverage rate as a covariate in the model. This allows any bias caused by differences in coverage rate to be estimated empirically and for the model to borrow strength based on this covariate. Secondly, the cancer registry in Iran is conducted mostly via a pathology-based system, which is less efficient than population-based registration. Thirdly, although we have considered the completeness of cancer registry by SSI registry, information on a small proportion of patients not supported by SSI is still absent in our models. Fourthly, the most recent data source is from 2010; this underlines the need for publication of more detailed and up-to-date information.

To our knowledge, this study is the first subnational level analysis of breast cancer incidence and mortality in Iran, simultaneously using several administrative datasets and Bayesian spatial modelling to obtain province-level estimates between 2000 and 2010 and also addressing the incompleteness of the cancer registry. Our results highlight the high levels of heterogeneity across provinces in the levels of incidence and mortality rates of breast cancer in Iran and the need for a comprehensive and effective plan to control breast cancer which takes into account subnational variability. These differences emphasise the urgent need to improve not only access to diagnosis but also access to treatment to contain breast cancer associated mortality in the most deprived areas and reduce inequalities.

## Conclusions

In conclusion, our findings showed that breast cancer incidence has increased over time in Iran, while mortality has decreased, but with lower incidence in the most deprived provinces possibly due to underdiagnosis or late-stage diagnosis. Although the mortality rate is still higher in wealthier provinces, the larger reduction observed over time in these provinces suggests a possible reversal in coming years, with the poorest provinces having higher levels of mortality. Improvements in prevention, access, and quality of screening procedures are needed to improve early diagnosis in the most deprived areas. The study also highlights the need for an improved cancer registry for breast cancer incidence monitoring to ensure the data can be actionable.

## Supporting information

S1 Appendix Bayesian Poisson spatial model. (DOCX)

S1 Table Summary table of covariates. (DOCX)

S1 Fig Map of covariates by time: cancer registry completeness percentage (a), female urbanization percentage (b), female mean years of schooling (c), and wealth index (d) (the last one is not used as covariate in the model but its correlation is checked with incidence and mortality rates).
(DOCX)

S2 Fig Age-standardised breast cancer incidence versus age-standardised mortality rate per 100,000 by three time intervals. (DOCX)

S3 Fig Box plots of age-standardised breast cancer incidence rate (a) and age-standardised breast cancer mortality rate (b). Diamond symbol shows the mean value.
(DOCX)

S1 Data (PDF)
