# Supplementary material for: Geographical and socioeconomic inequalities in female breast cancer incidence and mortality in Iran: A Bayesian spatial analysis of registry data
Source: PLoS One. 2021 Mar 17;16(3):e0248723. doi: 10.1371/journal.pone.0248723 (PMC7968648; doi:10.1371/journal.pone.0248723)
Supplement: S1 Appendix — (DOCX) [file pone.0248723.s001.docx]

**S1 Appendix. Bayesian Poisson spatial model**

We started from aggregated data by province level and considered them related over space (e.g. using a conditional autoregressive model). We used relevant predictive covariates from the Population and Housing Census. Our modelling has been performed using R and OpenBUGS and all age standardised (indirect method using total Iranian female rate in 2010) estimations were calculated based on the following Bayesian Poisson spatial model:

$$Y_{i}\sim Poisson \left( E_{i}\lambda_{i} \right)$$

$$\log\left( \lambda_{i} \right)= \alpha+ \beta_{yos}{YOS}_{i}+ \beta_{urb}{URB}_{i}+ \beta_{comp}{COMP}_{i}+ U_{i}+ \varepsilon_{i}$$

$Y_{i}$ : breast Cancer Count by province (i), No.province = 31

$E_{i}:$ the expected number of cases by province (i)

$\lambda_{i}:$ relative risk parameters by province (i)

$\alpha$ : intercept

${YOS}_{i}$ : female mean years of Schooling

${URB}_{i}$ : female urbanisation percentage

${COMP}_{i}$ : cancer registry completeness percentage

$U_{i}$ : spatially structured random terms of the complete Bayesian model

$\varepsilon_{i}$ : residual for province (i)

Based on this information, the posterior breast cancer incidence has been estimated in provinces level using the Bayesian Poisson spatial method.
