# Supplementary material for: Geographical and socioeconomic inequalities in female breast cancer incidence and mortality in Iran: A Bayesian spatial analysis of registry data
Source: PLoS One. 2021 Mar 17;16(3):e0248723. doi: 10.1371/journal.pone.0248723 (PMC7968648; doi:10.1371/journal.pone.0248723)
Supplement: S1 Table — (DOCX) [file pone.0248723.s002.docx]

| **year : 2000-2003** |  |  |  |  |
| --- | --- | --- | --- | --- |
|  | **Mean** | **Std. Dev.** | **Min** | **Max** |
| Female urbanization percentage | 51.3 | 12.6 | 36.8 | 91.7 |
| Female mean years of schooling | 2.5 | 0.6 | 1.7 | 4.0 |
| Cancer registry completeness | 0.3 | 0.2 | 0.1 | 0.7 |
| **year : 2004-2007** |  |  |  |  |
| Female urbanization percentage | 53.0 | 12.4 | 37.7 | 92.7 |
| Female mean years of schooling | 3.0 | 0.7 | 1.8 | 4.8 |
| Cancer registry completeness | 0.4 | 0.1 | 0.2 | 0.7 |
| **year : 2008-2010** |  |  |  |  |
| Female urbanization percentage | 54.3 | 12.3 | 38.5 | 93.5 |
| Female mean years of schooling | 3.5 | 0.8 | 2.0 | 5.7 |
| Cancer registry completeness | 0.7 | 0.1 | 0.4 | 0.8 |

S1 Table. Summary table of covariates
