# Supplementary material for: Geographical and socioeconomic inequalities in female breast cancer incidence and mortality in Iran: A Bayesian spatial analysis of registry data
Source: PLoS One. 2021 Mar 17;16(3):e0248723. doi: 10.1371/journal.pone.0248723 (PMC7968648; doi:10.1371/journal.pone.0248723)
Supplement: S1 Fig — Map of covariates by time: cancer registry completeness percentage (a), female urbanization percentage (b), female mean years of schooling (c), and wealth index (d) (the last one is not used as covariate in the model but its correlation is checked with incidence and mortality rates). (DOCX) [file pone.0248723.s003.docx]

**a b**


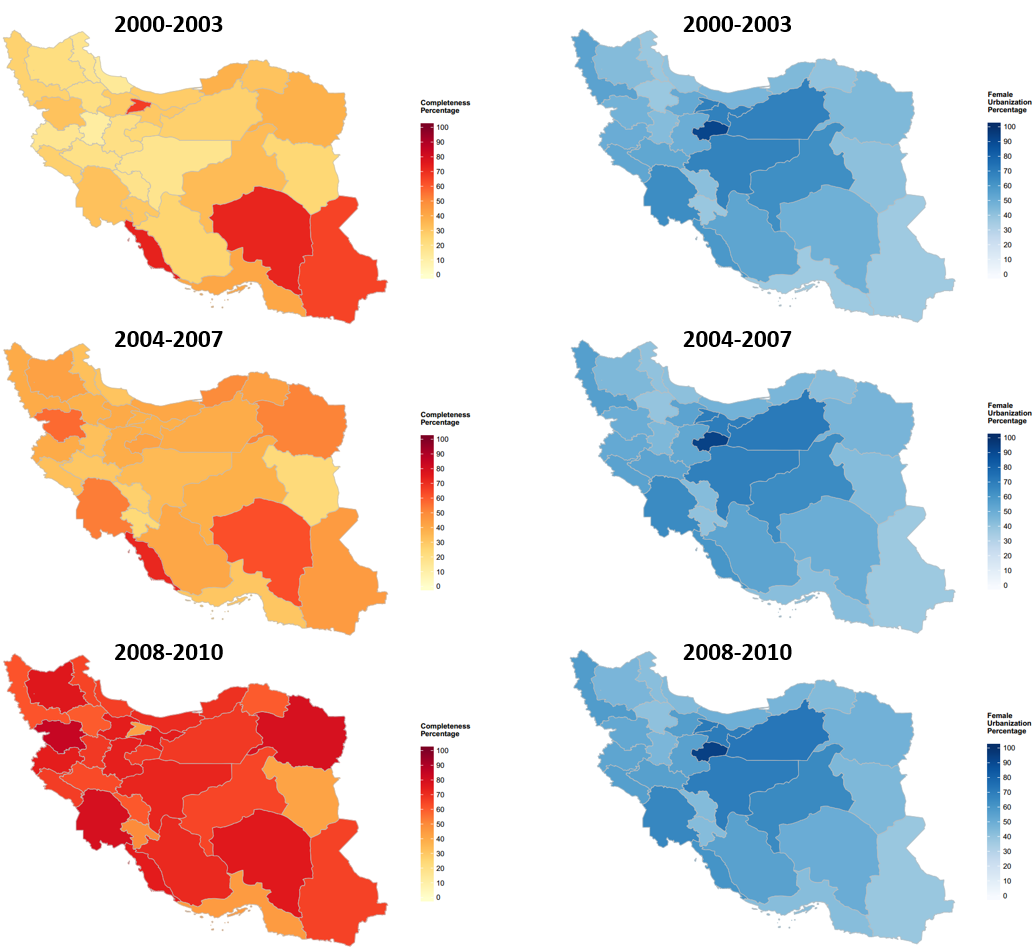


**c d**


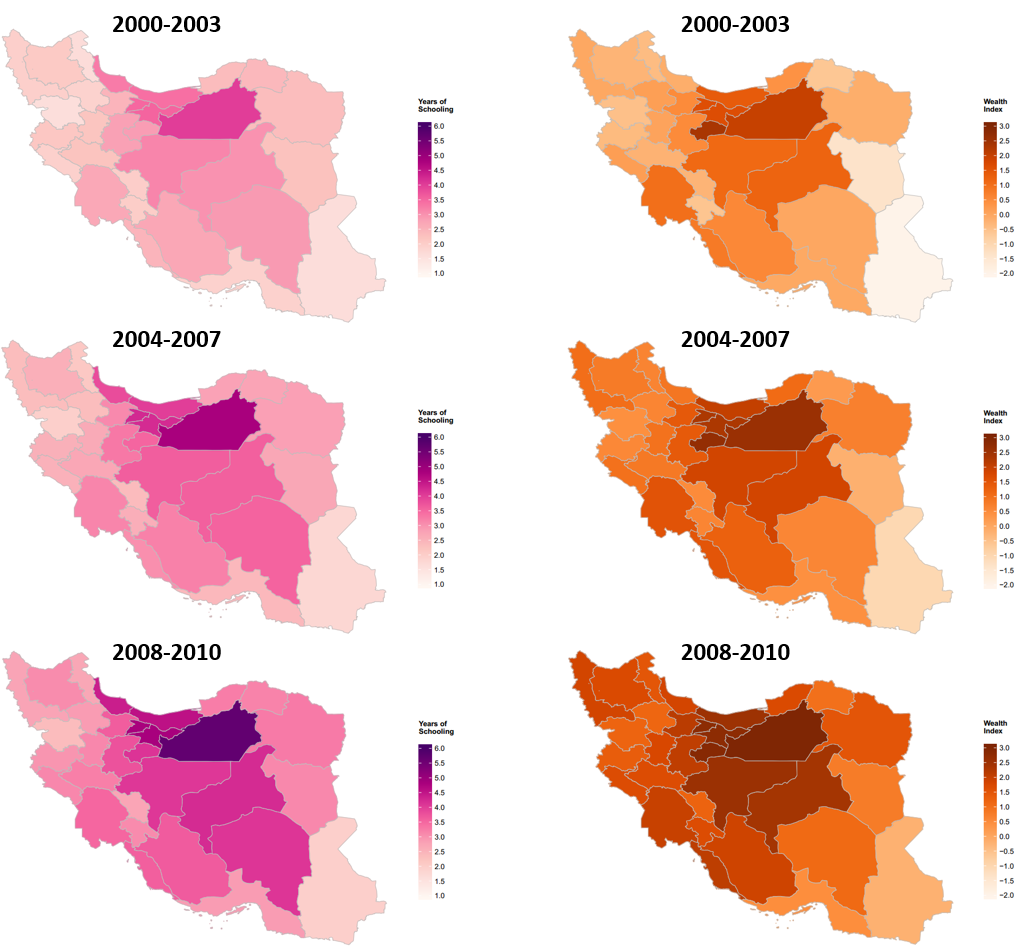


S1 Fig. Map of covariates by time: cancer registry completeness percentage (a), female urbanization percentage (b), female mean years of schooling (c), and wealth index (d) (the last one is not used as covariate in the model but its correlation is checked with incidence and mortality rates)
