# Supplementary material for: Geographical and socioeconomic inequalities in female breast cancer incidence and mortality in Iran: A Bayesian spatial analysis of registry data
Source: PLoS One. 2021 Mar 17;16(3):e0248723. doi: 10.1371/journal.pone.0248723 (PMC7968648; doi:10.1371/journal.pone.0248723)
Supplement: S2 Fig — (DOCX) [file pone.0248723.s004.docx]

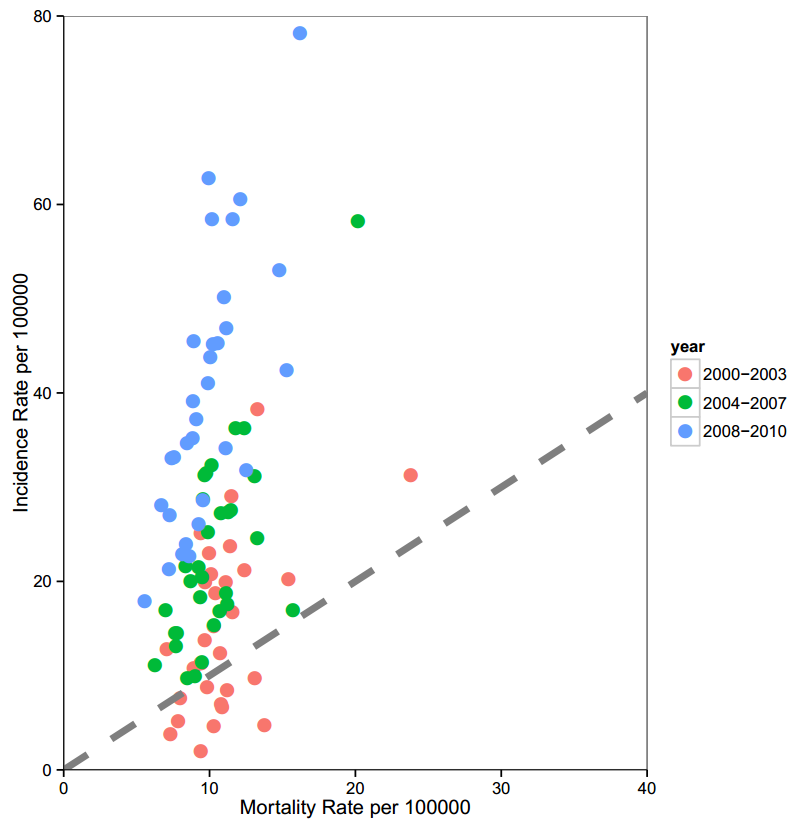


S2 Fig. Age-standardised breast cancer incidence versus age-standardised mortality rate per 100,000 by three time intervals
