# Supplementary material for: Geographical and socioeconomic inequalities in female breast cancer incidence and mortality in Iran: A Bayesian spatial analysis of registry data
Source: PLoS One. 2021 Mar 17;16(3):e0248723. doi: 10.1371/journal.pone.0248723 (PMC7968648; doi:10.1371/journal.pone.0248723)
Supplement: S3 Fig — Box plots of age-standardised breast cancer incidence rate (a) and age-standardised breast cancer mortality rate (b). Diamond symbol shows the mean value. (DOCX) [file pone.0248723.s005.docx]

**a**


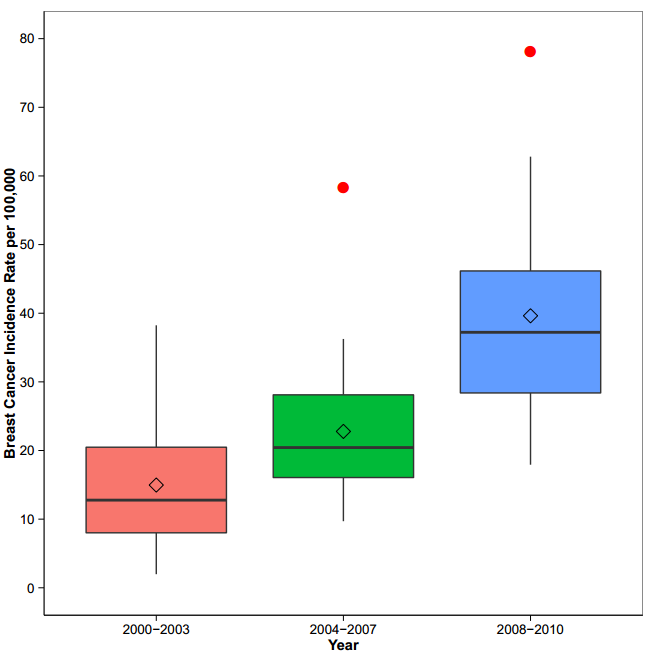


**b**


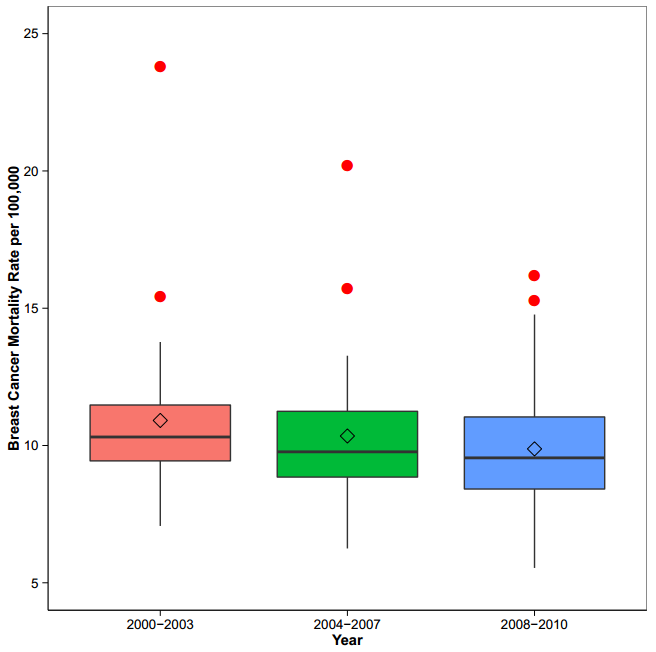


S3 Fig. Box plots of age-standardised breast cancer incidence rate (a) and age-standardised breast cancer mortality rate (b). Diamond symbol shows the mean value.
