# Supplementary material for: Geographical and socioeconomic inequalities in female breast cancer incidence and mortality in Iran: A Bayesian spatial analysis of registry data
Source: PLoS One. 2021 Mar 17;16(3):e0248723. doi: 10.1371/journal.pone.0248723 (PMC7968648; doi:10.1371/journal.pone.0248723)
Supplement: S1 Data — (PDF) [file pone.0248723.s006.pdf]

Year	Province	Female population	Breast cancer count	Expected number newcases	Mortality count	Expected number mortality	Female urbanization (%)	Female mean years of schooling	Wealth index	Cancer registry completeness	Raw incidence rate (per 100,000)	Estimated incidence rate (per 100,000)	Incidence lower limit (per 100,000)	Incidence upper limit (per 100,000)	Raw mortality rate (per 100,000)	Estimated mortality rate (per 100,000)	Mortality lower limit (per 100,000)	Mortality upper limit (per 100,000)
2000-2003	Markazi	958385	79	476.2	105	108.7	50.0	2.8	0.5	0.2	8.2	8.4	6.1	11.2	11.0	11.2	8.6	14.2
2000-2003	Gilan	1972486	393	991.7	219	220.9	40.2	3.3	-0.1	0.1	19.9	19.9	17.2	22.7	11.1	11.1	9.2	13.2
2000-2003	Mazandaran	2220292	275	1093.1	236	234.3	46.5	3.4	1.4	0.3	12.4	12.4	10.4	14.6	10.6	10.7	8.9	12.7
2000-2003	Azarbaijan,East	2589279	251	1285.9	347	281.0	43.6	2.1	-0.3	0.2	9.7	9.7	8.1	11.4	13.4	13.1	11.2	15.1
2000-2003	Azarbaijan,West	1823828	206	888.8	168	189.2	54.7	2.0	0.0	0.3	11.3	11.3	9.2	13.5	9.2	9.4	7.6	11.5
2000-2003	Kermanshah	1269842	254	618.2	123	129.4	50.1	2.2	-0.4	0.2	20.0	19.9	16.5	23.5	9.7	9.7	7.5	12.1
2000-2003	Khuzestan	2441421	615	1159.5	225	238.1	63.9	2.7	1.0	0.3	25.2	25.2	22.4	28.1	9.2	9.4	7.8	11.2
2000-2003	Fars	2796735	644	1360.3	277	291.3	53.8	2.7	0.6	0.3	23.0	23.0	20.5	25.5	9.9	10.0	8.4	11.6
2000-2003	Kerman	1483006	309	717.9	149	154.5	48.7	2.9	0.0	0.7	20.8	20.7	17.5	24.1	10.1	10.1	8.0	12.4
2000-2003	Khorasan,razavi	3535945	593	1730.8	414	374.8	44.9	2.4	-0.1	0.4	16.8	16.7	14.9	18.7	11.7	11.6	10.1	13.2
2000-2003	Isfahan	3155581	918	1546.4	359	338.5	67.8	3.2	1.1	0.2	29.1	29.1	26.5	31.8	11.4	11.5	9.9	13.2
2000-2003	Sistan and Baluchistan	1003492	37	472.5	72	96.8	36.8	1.7	-2.0	0.7	3.7	3.8	2.2	5.6	7.2	7.3	5.2	9.7
2000-2003	Kordestan	921461	100	449.6	80	95.5	47.8	1.7	-0.5	0.3	10.9	10.8	7.9	14.0	8.7	8.9	6.5	11.6
2000-2003	Hamadan	1199978	104	597.4	117	133.0	43.4	2.2	0.1	0.1	8.7	8.7	6.5	11.3	9.8	9.8	7.6	12.3
2000-2003	Chahar Mahal and Bakhtiari	515780	39	249.6	38	53.4	42.1	2.0	-0.3	0.2	7.6	7.6	4.5	11.3	7.4	8.0	5.0	11.3
2000-2003	Lorestan	1035170	143	499.6	99	105.4	53.0	2.2	-0.2	0.2	13.8	13.8	10.7	17.1	9.6	9.7	7.3	12.3
2000-2003	Ilam	311406	12	147.7	35	30.3	53.2	2.0	0.2	0.3	3.9	4.6	1.9	8.4	11.2	10.3	6.1	15.1
2000-2003	Kohgiluyeh and Boyer_Ahmad	335122	16	156.9	26	32.5	38.3	2.0	-0.6	0.3	4.8	5.2	2.1	9.0	7.8	7.9	4.5	11.9
2000-2003	Bushehr	510875	97	245.2	53	50.8	59.4	2.5	0.8	0.7	19.0	18.8	13.7	24.5	10.4	10.4	7.1	14.3
2000-2003	Zanjan	637071	73	314.9	60	69.2	38.2	1.9	0.2	0.2	11.5	11.3	7.9	15.1	9.4	9.5	6.6	12.7
2000-2003	Semnan	413840	87	205.1	43	46.1	68.6	4.0	2.0	0.3	21.0	21.2	15.5	27.8	10.4	12.4	8.2	17.2
2000-2003	Yazd	620536	239	306.4	83	69.8	63.0	3.0	1.2	0.3	38.5	38.2	31.6	45.4	13.4	13.3	9.7	17.2
2000-2003	Hormozgan	709087	92	332.9	46	70.7	37.3	1.9	0.0	0.4	13.0	12.8	9.3	16.6	6.5	7.1	4.7	9.7
2000-2003	Tehran	8010460	2506	3880.1	1926	809.0	68.4	3.5	1.6	0.3	31.3	31.3	29.6	33.0	24.0	23.8	22.3	25.3
2000-2003	Ardabil	793074	55	389.4	90	84.0	38.7	1.7	-0.4	0.2	6.9	7.0	4.7	9.7	11.4	10.8	7.9	14.0
2000-2003	Qom	629107	126	298.9	96	63.3	91.7	2.9	2.3	0.2	20.0	20.2	15.6	25.4	15.3	15.4	11.4	19.9
2000-2003	Qazvin	725750	175	350.7	85	75.4	51.7	2.5	0.5	0.3	24.1	23.8	18.9	29.1	11.7	11.4	8.4	14.7
2000-2003	Golestan	1032209	159	496.8	109	102.3	44.9	2.4	0.4	0.4	15.4	15.3	12.0	18.7	10.6	10.3	7.9	13.1
2000-2003	Khorasan,North	512754	6	253.0	47	53.4	40.5	2.5	-0.6	0.3	1.2	2.0	0.6	3.9	9.2	9.4	6.2	13.1
2000-2003	Khorasan,South	414839	26	209.9	46	49.2	41.6	2.3	-1.3	0.3	6.3	6.6	3.4	10.4	11.1	10.9	7.0	15.2
2000-2003	Alborz	1250970	56	593.3	173	119.2	68.8	3.5	1.6	0.7	4.5	4.8	3.2	6.6	13.8	13.8	11.0	16.7
2004-2007	Markazi	1094759	204	543.7	120	124.1	52.7	3.3	1.4	0.4	18.6	18.8	15.4	22.5	11.0	11.1	8.7	13.8
2004-2007	Gilan	2246817	615	1140.2	239	255.2	41.6	3.8	0.9	0.3	27.4	27.3	24.3	30.4	10.6	10.8	9.0	12.7
2004-2007	Mazandaran	2556285	699	1276.7	286	274.2	47.8	4.0	2.0	0.4	27.3	27.3	24.5	30.2	11.2	11.3	9.6	13.1
2004-2007	Azarbaijan,East	2947150	815	1465.2	348	323.4	45.0	2.6	0.8	0.4	27.7	27.5	24.9	30.3	11.8	11.5	9.8	13.2
2004-2007	Azarbaijan,West	2124786	462	1041.8	172	224.1	56.4	2.4	1.0	0.4	21.7	21.7	18.9	24.5	8.1	8.4	6.7	10.1
2004-2007	Kermanshah	1457180	370	718.9	147	152.4	51.8	2.6	0.6	0.4	25.4	25.2	21.7	28.9	10.1	9.9	7.8	12.2
2004-2007	Khuzestan	2827766	893	1352.8	274	277.1	64.8	3.2	1.5	0.5	31.6	31.5	28.6	34.5	9.7	9.8	8.2	11.4
2004-2007	Fars	3253002	1054	1598.5	329	343.0	54.3	3.2	1.2	0.4	32.4	32.3	29.6	35.1	10.1	10.1	8.7	11.7
2004-2007	Kerman	1783629	362	869.3	167	186.5	49.9	3.6	0.6	0.6	20.3	20.4	17.5	23.5	9.4	9.5	7.6	11.5
2004-2007	Khorasan,razavi	4124621	1293	2027.6	402	438.5	46.6	2.8	0.7	0.5	31.4	31.3	28.9	33.7	9.8	9.7	8.4	11.0
2004-2007	Isfahan	3686715	1339	1822.8	430	397.4	68.9	3.6	1.8	0.3	36.3	36.3	33.6	39.0	11.7	11.8	10.3	13.4
2004-2007	Sistan and Baluchistan	1202959	132	566.3	74	115.8	37.7	1.8	-1.0	0.5	11.0	11.1	8.7	13.8	6.2	6.3	4.5	8.2
2004-2007	Kordestan	1065953	153	520.8	80	111.6	50.0	2.0	0.4	0.6	14.4	14.6	11.5	17.9	7.5	7.7	5.6	9.9
2004-2007	Hamadan	1347025	247	671.8	125	150.4	44.9	2.7	1.0	0.3	18.3	18.3	15.2	21.6	9.3	9.4	7.3	11.7
2004-2007	Chahar Mahal and Bakhtiari	603319	87	292.7	43	62.9	43.1	2.4	0.5	0.3	14.4	14.5	10.6	18.9	7.1	7.8	5.1	10.8
2004-2007	Lorestan	1206427	242	583.9	100	123.6	54.4	2.7	0.8	0.3	20.1	20.0	16.6	23.7	8.3	8.7	6.6	10.9
2004-2007	Ilam	373264	62	177.3	47	36.5	54.9	2.6	1.0	0.3	16.6	16.8	11.5	22.8	12.6	10.7	6.7	15.3
2004-2007	Kohgiluyeh and Boyer_Ahmad	404545	37	190.3	41	39.5	40.7	2.6	0.6	0.2	9.2	10.0	6.2	14.3	10.1	9.0	5.7	12.9
2004-2007	Bushehr	593520	171	285.8	57	59.4	60.5	3.0	1.5	0.7	28.8	28.7	22.9	35.1	9.6	9.5	6.6	13.0
2004-2007	Zanjan	735693	95	362.0	51	80.3	39.7	2.4	0.6	0.4	12.9	13.1	9.7	17.0	6.9	7.7	5.3	10.3
2004-2007	Semnan	473262	112	236.3	52	52.6	71.3	4.8	2.6	0.4	23.7	24.6	18.6	31.1	11.0	13.3	9.1	17.8
2004-2007	Yazd	720670	264	357.0	88	80.9	64.2	3.6	1.8	0.4	36.6	36.2	30.3	42.6	12.2	12.4	9.2	15.8
2004-2007	Hormozgan	853141	146	403.6	54	85.1	42.3	2.5	0.4	0.3	17.1	16.9	13.3	21.0	6.3	7.0	4.8	9.4
2004-2007	Tehran	9493876	5539	4664.9	1934	973.6	69.9	4.2	2.3	0.4	58.3	58.3	56.1	60.5	20.4	20.2	18.9	21.5
2004-2007	Ardabil	913091	102	447.4	92	97.9	40.6	2.2	0.6	0.3	11.2	11.4	8.5	14.6	10.1	9.5	7.0	12.3
2004-2007	Qom	747232	231	357.7	94	75.2	92.7	3.5	2.7	0.4	30.9	31.2	25.7	37.1	12.6	13.1	9.8	16.9
2004-2007	Qazvin	859046	149	416.2	100	89.4	54.3	3.1	1.4	0.4	17.3	17.6	13.9	21.7	11.6	11.2	8.4	14.3
2004-2007	Golestan	1207680	261	586.4	115	121.0	46.0	2.8	1.1	0.5	21.6	21.5	18.0	25.3	9.5	9.3	7.0	11.6
2004-2007	Khorasan,North	588353	51	292.0	48	62.1	42.2	2.8	0.2	0.4	8.7	9.7	6.5	13.4	8.2	8.5	5.8	11.6

Year	Province	Female population	Breast cancer count	Expected number newcases	Mortality count	Expected number mortality	Female urbanization (%)	Female mean years of schooling	Wealth index	Cancer registry completeness	Raw incidence rate (per 100,000)	Estimated incidence rate (per 100,000)	Incidence lower limit (per 100,000)	Incidence upper limit (per 100,000)	Raw mortality rate (per 100,000)	Estimated mortality rate (per 100,000)	Mortality lower limit (per 100,000)	Mortality upper limit (per 100,000)
2004-2007	Khorasan,South	472874	72	238.3	49	56.1	43.2	2.7	-0.2	0.2	15.2	15.3	10.8	20.5	10.4	10.3	7.0	14.2
2004-2007	Alborz	1609669	267	774.2	260	155.8	70.1	4.3	2.3	0.4	16.6	17.0	14.2	19.9	16.2	15.7	13.1	18.5
2008-2010	Markazi	932043	438	464.9	104	106.1	55.3	3.8	2.1	0.7	47.0	46.9	40.9	53.1	11.2	11.2	8.5	14.0
2008-2010	Gilan	1880673	852	968.4	194	217.0	44.1	4.4	1.8	0.7	45.3	45.3	41.1	49.7	10.3	10.6	8.6	12.6
2008-2010	Mazandaran	2165384	1084	1099.3	233	237.1	49.2	4.5	2.5	0.7	50.1	50.1	46.0	54.4	10.8	11.0	9.1	12.9
2008-2010	Azarbaijan,East	2493424	1131	1245.1	265	275.5	46.2	3.1	1.7	0.8	45.4	45.2	41.5	48.9	10.6	10.2	8.6	12.0
2008-2010	Azarbaijan,West	1831279	494	904.7	122	194.3	57.7	2.8	1.8	0.6	27.0	27.0	23.8	30.4	6.7	7.3	5.6	9.0
2008-2010	Kermanshah	1235084	509	617.9	129	131.7	53.7	3.0	1.3	0.7	41.2	41.0	36.1	46.1	10.4	9.9	7.7	12.3
2008-2010	Khuzestan	2444030	1542	1174.8	243	240.7	65.9	3.5	2.0	0.8	63.1	62.8	58.4	67.3	9.9	9.9	8.3	11.7
2008-2010	Fars	2820630	1655	1394.9	290	299.1	55.2	3.6	1.9	0.7	58.7	58.4	54.5	62.5	10.3	10.2	8.6	11.8
2008-2010	Kerman	1579360	612	771.0	132	164.6	50.2	4.1	1.1	0.8	38.8	39.1	34.8	43.6	8.4	8.9	7.0	10.8
2008-2010	Khorasan,razavi	3562985	1622	1756.6	318	379.9	47.9	3.3	1.5	0.8	45.5	45.5	42.4	48.7	8.9	8.9	7.6	10.3
2008-2010	Isfahan	3184656	1864	1587.6	365	346.0	69.8	4.1	2.5	0.7	58.5	58.4	54.7	62.3	11.5	11.6	10.0	13.2
2008-2010	Sistan and Baluchistan	1052000	184	489.8	56	100.1	38.5	2.0	-0.2	0.7	17.5	17.9	14.5	21.6	5.3	5.5	3.8	7.4
2008-2010	Kordestan	903238	297	445.0	63	95.4	52.2	2.4	1.2	0.8	32.9	33.1	28.0	38.3	7.0	7.4	5.2	9.7
2008-2010	Hamadan	1131486	423	567.6	103	127.5	45.9	3.1	1.7	0.7	37.4	37.2	32.4	42.3	9.1	9.1	7.0	11.4
2008-2010	Chahar Mahal and Bakhtiari	515362	175	251.4	37	54.2	43.7	2.8	1.2	0.6	34.0	33.2	26.6	40.4	7.2	7.6	4.9	10.7
2008-2010	Lorestan	1038307	361	505.2	81	107.4	55.8	3.2	1.6	0.6	34.8	34.7	29.8	39.8	7.8	8.4	6.3	10.8
2008-2010	Ilam	321777	89	154.1	36	31.8	56.4	3.1	1.7	0.7	27.7	28.6	21.1	37.0	11.2	9.6	5.9	14.0
2008-2010	Kohgiluyeh and Boyer_Ahmad	353925	79	167.5	33	34.6	43.2	3.1	1.6	0.5	22.3	22.9	16.4	30.0	9.3	8.1	4.8	11.9
2008-2010	Bushehr	523678	229	251.1	55	52.2	61.1	3.6	2.1	0.7	43.7	43.8	36.1	51.9	10.5	10.1	6.9	13.6
2008-2010	Zanjan	628676	128	309.2	37	68.4	41.0	2.9	1.2	0.6	20.4	21.3	16.5	26.4	5.9	7.2	4.8	9.9
2008-2010	Semnan	401446	206	202.1	50	44.9	72.6	5.7	3.0	0.7	51.3	53.0	43.3	63.3	12.5	14.8	10.2	19.9
2008-2010	Yazd	620815	382	307.7	75	69.1	65.3	4.2	2.4	0.6	61.5	60.5	52.2	69.3	12.1	12.1	8.9	15.6
2008-2010	Hormozgan	766548	221	361.3	47	75.2	42.9	2.9	0.5	0.4	28.8	28.1	23.1	33.5	6.1	6.7	4.4	9.1
2008-2010	Tehran	8282025	6476	4129.7	1354	870.9	70.7	4.8	2.8	0.7	78.2	78.2	75.5	80.9	16.4	16.2	15.0	17.4
2008-2010	Ardabil	771241	170	379.5	72	82.7	42.4	2.7	1.5	0.6	22.0	22.6	18.2	27.5	9.3	8.6	6.1	11.4
2008-2010	Qom	655273	200	315.9	76	65.9	93.5	4.1	2.9	0.7	30.5	31.8	25.9	38.0	11.6	12.5	9.2	16.3
2008-2010	Qazvin	741687	248	361.9	89	77.7	56.8	3.6	2.1	0.7	33.4	34.1	28.5	40.2	12.0	11.1	8.2	14.3
2008-2010	Golestan	1051342	370	512.3	97	105.6	46.7	3.3	1.7	0.7	35.2	35.2	30.3	40.3	9.2	8.8	6.7	11.2
2008-2010	Khorasan,North	500885	115	249.4	42	53.2	43.7	3.2	1.0	0.6	23.0	24.0	18.4	30.2	8.4	8.4	5.4	11.6
2008-2010	Khorasan,South	391737	104	197.5	38	46.0	45.1	3.1	0.8	0.4	26.6	26.0	19.7	33.2	9.7	9.3	5.9	13.3
2008-2010	Alborz	1495417	637	725.6	241	146.6	71.0	4.8	2.8	0.4	42.6	42.4	37.9	47.2	16.1	15.3	12.6	18.2
